# Exogenous Melatonin Application Enhances *Rhizophagus irregularis* Symbiosis and Induces the Antioxidant Response of *Medicago truncatula* Under Lead Stress

**DOI:** 10.3389/fmicb.2020.00516

**Published:** 2020-04-15

**Authors:** Xiangyu Zhang, Huijuan Zhang, Haoqiang Zhang, Ming Tang

**Affiliations:** ^1^State Key Laboratory of Soil Erosion and Dryland Farming on the Loess Plateau, College of Forestry, Northwest A&F University, Yangling, China; ^2^State Key Laboratory for Conservation and Utilization of Subtropical Agro-Bioresources, Lingnan Guangdong Laboratory of Modern Agriculture, Guangdong Key Laboratory for Innovative Development and Utilization of Forest Plant Germplasm, College of Forestry and Landscape Architecture, South China Agricultural University, Guangzhou, China

**Keywords:** arbuscular mycorrhizal fungus, lead uptake, melatonin synthesis, antioxidant response, symbiosis

## Abstract

Melatonin is a new kind of plant growth regulator. The aim of this study was to figure out the effect of melatonin on arbuscular mycorrhizal (AM) symbiosis and heavy metal tolerance. A three-factor experiment was conducted to determine the effect of melatonin application on the growth, AM symbiosis, and stress tolerance of *Medicago truncatula*. A two-factor (AM inoculation and Pb stress) experiment was conducted to determine the effect of AM fungus on melatonin accumulation under Pb stress. AM plants under Pb stress had a higher melatonin accumulation than non-mycorrhizal (NM) plants under Pb stress. Acetylserotonin methyltransferase (ASMT) is the enzymatic reaction of the last step in melatonin synthesis. The accumulation of melatonin may be related to the expression of *MtASMT*. Melatonin application increased the relative expression of *MtPT4* and AM colonization in AM plants. Melatonin application decreased Pb uptake with and without AM inoculation. Both melatonin application and AM inoculation improved *M. truncatula* growth and increased antioxidant response with Pb stress. These results indicated that melatonin application has positive effects on AM symbiosis and Pb stress tolerance under Pb stress. AM inoculation improve melatonin synthesis capacity under Pb stress. Melatonin application may improve AM plant growth by enhancing AM symbiosis, stimulating antioxidant response, and inhibiting Pb uptake.

## Introduction

With social and economic development, soil heavy metal pollution has produced a series of problems in China (Li et al., 2014). Anthropogenic activities such as mining and smelting are the primary sources of soil heavy metal pollution (Yang et al., 2018). Lead (Pb), a toxic element in plants, enters the soil mainly through lead-zinc mining (Yabe et al., 2018). More than 800,000 tons of Pb have been released into the environment globally, and most Pb has accumulated in the soil environment, where it negatively affects plant growth and development (Yang et al., 2018).

Melatonin, *N*-acetyl-5-methoxytryptamine, was first isolated and identified from the bovine pineal gland (Lerner et al., 1960). Melatonin is a tryptophan-derived molecule that acts as an antioxidant under abiotic stress (Burkhardt et al., 2001). In 1995, melatonin was detected in the edible plants tomato and banana, which suggests that melatonin is ubiquitous in plants (Dubbels et al., 1995). A recent study identified melatonin receptor 1 in *Arabidopsis thaliana*, serving as strong evidence that melatonin might be a new growth regulator in plants involved in plant growth and stress tolerance (Wei et al., 2018; Arnao and Hernandez-Ruiz, 2019). Melatonin synthesis in plants is primarily divided into two steps that involve four enzymes, tryptophan decarboxylase (TDC), tryptamine 5-hydroxylase (T5H), serotonin *N*-acetyltransferase (SNAT), and acetylserotonin methyltransferase (ASMT) (Back et al., 2016; Lee et al., 2017). The first step of melatonin synthesis in plants is the transformation of tryptophan to serotonin through TDC and T5H activity. The second step of melatonin synthesis is the transformation of serotonin to melatonin through SNAT and ASMT (Back et al., 2016). ASMT, a terminal enzyme in melatonin synthesis, is the rate-limiting step during melatonin synthesis (Park et al., 2013). Exogenous melatonin application increases plant survival under conditions of heavy metal toxicity through improving antioxidant capacity and enhancing the levels of protective molecules (Li et al., 2016; Gu et al., 2017). Heavy metal-stressed plants regulate the expression of melatonin synthesis-related genes, such as *OsASMT* (Byeon et al., 2015), to enhance heavy metal tolerance. Therefore, melatonin-rich plants or plants to which melatonin has been exogenously applied have a higher potential for the improvement of plant growth and stress tolerance (Tan and Reiter, 2015; Tang et al., 2018).

Arbuscular mycorrhizal (AM) symbiosis is a mutualistic endosymbiosis between AM fungi and terrestrial plants (Javot et al., 2007). AM fungi are abundant in heavy metal-contaminated areas, such as areas of lead-zinc mining (Yang et al., 2015). MtPT4 is the low-affinity phosphate (Pi) transporter in *Medicago truncatula* located in arbuscule-colonized cells that are specifically induced in mycorrhizal roots (Javot et al., 2007). The expression of *MtPT4* is used to determine the symbiotic state of the colonized root system (Isayenkov et al., 2004). AM fungi were shown to improve stress tolerance by stimulating the synthesis of the endogenous growth regulator jasmonate (Sánchez-Romera et al., 2016) and strigolactone (Aroca et al., 2013). However, how mycorrhizal plants regulate melatonin synthesis under heavy metal stress is unclear. Whether the simultaneous application of AM fungi and melatonin to a host plant improves its AM symbiosis, growth conditions, and heavy metal tolerance is unknown.

We hypothesized that AM inoculation enhances melatonin accumulation under Pb stress and that the application of melatonin would promote AM plant growth and Pb tolerance. Therefore, the purpose of this study was to determine the effect of melatonin application on AM symbiosis, growth, and Pb tolerance through evaluating AM colonization, Pb levels, the antioxidant response, and proline accumulation. Moreover, the difference of melatonin accumulation between mycorrhizal and non-mycorrhizal (NM) plants under Pb stress was determined to evaluate the effect of AM inoculation on melatonin production. This study provides a new evidence for the role of melatonin in AM plants.

## Materials and Methods

### Plant Material, AM Fungal Inoculum, and Substrates

Seeds of *M. truncatula* (Jemalong A17) were kindly provided by Prof. Philipp Franken (Plant Physiology Department, Humboldt University of Berlin). Seeds were sterilized by concentrated sulfuric acid for 10 min and washed by sterile water 10 times. Sterilized seeds were placed in Petri dishes with 0.8% water agar at 4°C in the dark for 2 days, then at 26°C in the dark for 1 day, and finally at 26°C in the light (3000 lx) for 1 day. Uniformed seedlings were transplanted into the pot (10 cm diameter, 12 cm height), which contained 0.45 kg mixed substrates (sand: soil = 1:1). The sand was sieved through a 2-mm soil sieve and then washed with tap water until the supernatant was clear. After drying, the sand was sterilized in the oven at 170°C for 3 h. The soil was collected from the nursery garden of Northwest A&F University. The soil was sieved through a 2-mm soil sieve. The soil (loam, pH 8.2) contained 4.12 g kg^–1^ organic matter, 14.05 mg kg^–1^ Olsen phosphorus, 24.81 mg kg^–1^ available nitrogen, and 55.14 mg kg^–1^ rapidly available potassium. Soils were sterilized in the autoclave at 121°C for 2 h. The AM inoculum of *Rhizophagus irregularis* (Bank of Glomales in China, No. BGC BJ09) consisted of the sandy substrate that contained spores (approximately 21 spores per gram), mycelia, and colonized root fragments. Each AM treatment was inoculation with 20 g of inoculum. Each NM treatment was inoculation with 20 g of sterilized inoculum (3 h in an oven at 170°C). The inoculum was provided by Beijing Academy of Agriculture and Forestry Sciences (Beijing, China).

### Experimental Design

Experiment 1 was performed as a two-factorial experiment using two Pb levels (0 and 800 mg kg^–1^ substrate) and two AM fungi treatment conditions (with and without AM fungi inoculation). Each pot contained one *M. truncatula* seedlings and 450 g of sterilized sand and soil (1:1 v:v). Each pot added 20 g of sterilized (NM treatment) or unsterilized inoculum (AM treatment). Therefore, each pot contained 470 g of substance. Each treatment consisted of four biological replicates, and each biological replicate consisted of four pots of seedlings. Fifty milliliters of a Pb solution (7.52 g L^–1^) was added to Pb-treated seedlings once after the seedlings had been cultivated for 2 weeks. A Pb stock solution was prepared with Pb(NO_3_)_2_. The NO_3_^–^ concentrations among all treatments were normalized by using the NO_3_^–^ salt of the relevant compound (4:1:1 HNO_3_:KNO_3_: NaNO_3_) to eliminate the effects of NO_3_^–^ from Pb(NO_3_)_2_ following the method of Wang et al. (2012). Plants treated with Pb suffered Pb stress for 12 weeks.

Experiment 2 was performed as a three-factorial experiment using two Pb levels (0 and 800 mg kg^–1^ substrate), two AM fungi treatment conditions (with and without AM fungi inoculation), and two melatonin treatment conditions (with and without exogenous melatonin application). Each pot contained one *M. truncatula* seedling and 450 g of sterilized sand and soil (1:1 v:v). Each treatment consisted of four biological replicates, and each biological replicate consisted of three pots of seedlings. Pb treatment is the same as experiment 1. Plants treated with Pb suffered Pb stress for 12 weeks. One hundred milliliters of a melatonin solution (10 μM melatonin) was added to the culture substance of melatonin-treated seedlings at 12 weeks post-colonization following the method of Antoniou et al. (2017). Melatonin treatment lasted for 2 weeks. To plants not treated with melatonin, 100 ml of ddH_2_O was applied.

Twenty milliliters of modified Hoagland’s nutrient solution [5 mM Ca(NO_3_)_2_, 5 mM KNO_3_, 2 mM MgSO_4_, 0.2 mM KH_2_PHO_4_, 46 μM H_3_BO_3_, 9 μM MnCl_2_, 0.8 μM ZnSO_4_, 0.3 μM CuSO_4_, 0.1 μM H_2_MoO_4_, and 18 μM FeNaEDTA] was added to each treatment of both experiment 1 and experiment 2 once a week. All *M. truncatula* seedlings were grown in the greenhouse with 28°C/24°C day/night temperature under 16 h daylight and 40–60% humidity. Water was supplied every day throughout plant growth to maintain soil moisture.

### Plant Sampling and Biomass Measurement

The seedlings from each treatment group (experiment 1 and experiment 2) were harvested 14 weeks post-colonization. The shoots were cut, and the roots were separated. Fresh shoot and root biomass were weighed. Parts of the roots were used to assess the effect of AM colonization. Parts of the samples were dried for Pb measurement. The remaining parts of the samples were ground to a powder using liquid nitrogen and stored at −80°C for further analysis.

### AM Colonization

The root samples were stained with trypan blue (0.12%) and assessed using the method of Hu et al. (2017). Decolorized root segments were placed parallel to the long axis of a slide and then covered with a transparent coverslip. Five slides were prepared for each sample. Another coverslip with a vertical line was placed over the transparent coverslip. All intersections between roots and the vertical line were counted. AM colonization was calculated as follows: (count number of hyphae, vesicles, and arbuscules)/total counted number.

### Melatonin Determination

Melatonin was extracted using an acetone–methanol method (Pape and Lüning, 2006). The powdered samples (0.1 g) were extracted in 5 ml of an extraction mixture (acetone:methanol:water = 89:10:1) in the dark, and trichloroacetic acid was used to precipitate protein. The extracts were centrifuged (12,000 × *g*, 4°C) for 15 min, and then the supernatants were used for measurements. A plant melatonin ELISA kit was used to evaluate the melatonin content following the manufacturer’s instructions (Shanghai Jiwei Biological Technology Co., Ltd., China).

### RNA Isolation and Quantitative Real-Time PCR (qRT-PCR) Analysis

The powered root samples were used for total RNA extraction by E.Z.N.ATM plant RNA kit (Omega Bio-Tek, Norcross, GA, United States). Each treatment consists of four biological replicates. The RNA quality of each sample was evaluated by 1% agarose gels stained with DuRed. RNA concentrations were determined by NanoDrop 2000 (Thermo Fisher Scientific, Pittsburgh, PA, United States). First-strand cDNA synthesis was obtained from 2 μg of total RNA using the PrimerScript^®^ First-strand cDNA Synthesis Kit (TaKaRa Bio, Dalian, China). Gene-specific primers for four melatonin synthesis genes, *MtPT4*, and *MtP5CS* were designed as described in Supplementary Table S1. The *M. truncatula* elongation factor 1-alpha gene (*MtEF-1*α, DQ282611.1) was used as an internal control (Jiang et al., 2018; Zeng et al., 2020). qRT-PCR was performed based on SYBR Green PCR and MIQE guidelines. CF96X Real-time PCR system (Bio-Rad, Hercules, CA, United States) was used to perform the qPCR experiments. The reaction volume was 20 μl containing 0.5 μl each gene-specific primer (10 μM), 2.0 μl of cDNA, 7 μl RNase-free H_2_O, and 10 μl SYBR Green PCR master mix (Roche Diagnostics, Basel, Switzerland). qPCR was performed under the following thermal cycles: 10 min at 95°C and 40 cycles of denaturation at 95°C for 15 s, annealing at 55°C for 15 s, extension at 72°C for 20 s, followed by heating from 60 to 95°C at a rate of 0.5°C per 10 s. The specificity of the primer pairs was indicated by the melting curve. The amplification efficiency of each primer pair was measured by the method of the standard curve.

### Measurement of the Pb Content

The dried root and leaf samples (0.05 g) were digested with 10 ml of HClO_4_ + HCl (4:1) at 300°C for 5 h. H_2_O_2_ was added after brown smoke was produced. The Pb content in the digested solution was determined by atomic absorption spectrometry (PinAAcle 500, United States) (Tüzen, 2003).

### Measurement of the Malonaldehyde (MDA) Content

MDA was extracted in 1 ml of 5% trichloroacetic acid from 0.1 g of powdered sample and centrifuged (12,000 × *g*) for 20 min. Thiobarbital acid was added to supernatant and then heated in boiling water bath for 30 min and centrifuged (5,000 × *g*) for 10 min. Supernatant was measured for absorbance at 450 nm, 532 nm, and 600 nm. MDA content was calculated by the method of Kumar and Knowles (1993).

### Measurement of Antioxidant Enzyme and P5C Reductase (P5CR) Activity

Powdered root samples were incubated with an enzyme extraction solution (50 mM potassium phosphate buffer, 1 mM EDTA, and 1% polyvinylpyrrolidone, 4°C) and centrifuged (14,000 × *g*) for 30 min at 4°C. The supernatant was used to determine the superoxide dismutase (SOD) and catalase (CAT) activity following the method of Beyer and Fridovich (1987). Ascorbate peroxidase (APX) activity was determined by the method of Nakano and Asada (1981). P5CR activity was determined by the method of Madan et al. (1995).

### Measurement of Proline and Flavone Content

The powdered root samples were extracted in 3% sulfosalicylic acid and centrifuged (10,000 × *g*) for 15 min. The supernatant was used to determine the proline content by using the method of Bates et al. (1973). The powdered root samples were extracted in 80% methyl ethanol (Zhishen et al., 1999). Rutin was used as a standard to determine the total flavone contents. The absorbance at 510 nm was measured.

### Statistical Analysis

Statistical analysis was performed using the SPSS 22.0 statistical program (SPSS Inc., Chicago, IL, United States). The data used for statistical analysis exhibited a normal distribution. The data in experiment 1 were analyzed using multifactor analysis of variance with two factors (AM fungi inoculation and Pb treatment) followed by Tukey HSD test when ANOVA indicated a significant difference. The data in experiment 2 were analyzed using multifactor analysis of variance with three factors (AM fungi inoculation, Pb treatment, and melatonin application) followed by Tukey HSD test when ANOVA indicated a significant difference. Correlation analysis was performed using Pearson’s test (*P* < 0.05).

## Results

### Melatonin Contents

**FIGURE 1:**
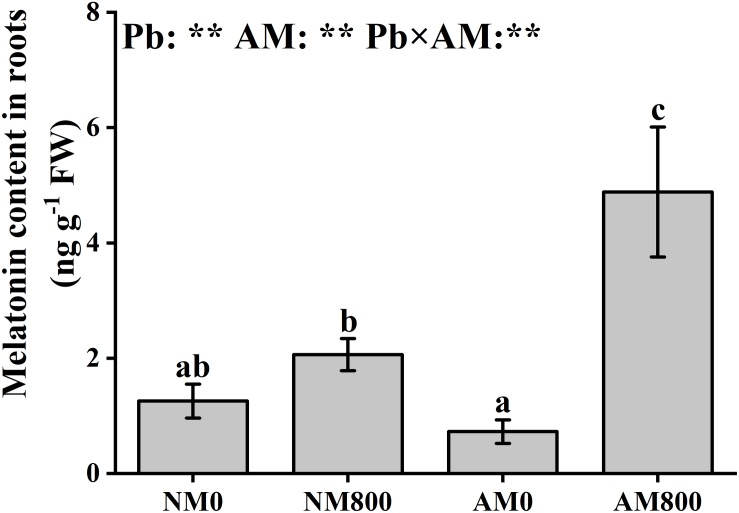
The melatonin content of mycorrhizal roots under Pb stress. The data are the means ± standard deviation (*n* = 4). Different letters above the columns indicate significant difference among the means of melatonin content by Tukey HSD’s test (*P* < 0.05), respectively. AM = AM inoculation; Pb = lead stress; NM0 = non-AM inoculation in the absence of Pb treatment; NM800 = non-AM inoculation with 800 mg kg^–1^ Pb treatment; AM0 = AM inoculation in the absence of Pb treatment; AM800 = AM inoculation with 800 mg kg^–1^ Pb treatment. Significant effect of two-way ANOVA: **P < 0.01; NS, not significant.

Arbuscular mycorrhizal inoculation did not affect the melatonin content in roots without Pb stress. With Pb stress, AM inoculation increased the melatonin content in the roots (Figure 1). Pb stress largely increased the melatonin content in both AM and NM roots compared to the melatonin content of the AM and NM roots of unstressed plants. AM inoculation and Pb stress had a significant effect on the root melatonin content.

### Expression of Melatonin Synthesis Genes

**FIGURE 2:**
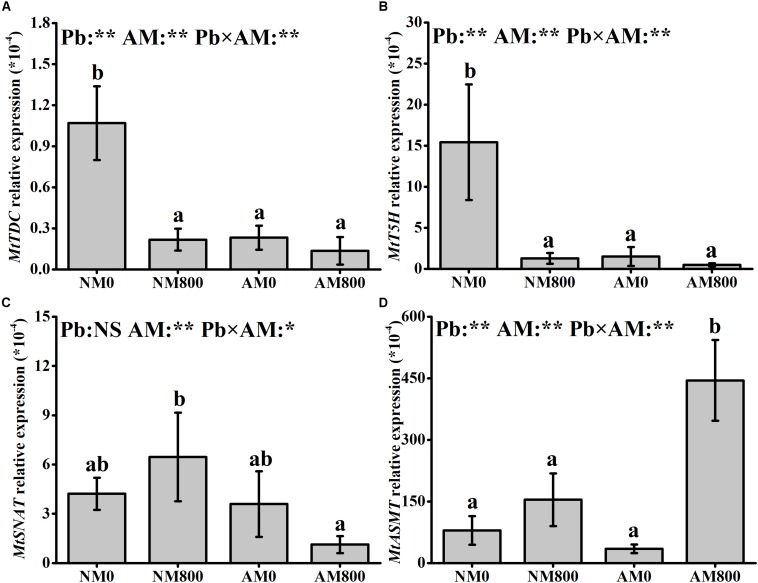
**(A–D)** The relative expression of *MtTDC*, *MtT5H*, *MtSNAT* and *MtASMT* of AM roots under Pb stress. The data are the means ± standard deviation (*n* = 4). Different letters above the columns indicate significant difference among the means of melatonin synthesis-related genes expression by Tukey HSD’s test (*P* < 0.05), *P < 0.05, **P < 0.01; NS, not significant. The abbreviation is consistent with the above.

Pb stress largely decreased (*P* < 0.05) the relative expression of *MtT5H* and *MtTDC* in NM roots (Figures 2A,B). AM inoculation decreased (*P* < 0.05) the relative expression of *MtT5H* and *MtTDC* in the absence of Pb stress. In AM roots, Pb stress upregulated (*P* < 0.05) the relative expression of *MtASMT* by 12-fold compared to the AM roots of unstressed plants (Figure 2D). In the absence of Pb stress, the relative expression of *MtSNAT* and *MtASMT* was not influenced by AM inoculation. In the presence of Pb stress, AM inoculation upregulated (*P* < 0.05) the relative expression of *MtASMT* by 2-fold but downregulated the relative expression of *MtSNAT* (Figure 2C). The relative expression of *MtASMT* was positively correlated (*MtASMT*: *r* = 0.976, *P* < 0.001) with melatonin content in the roots (Supplementary Figure S1).

### Biomass and AM Colonization

**FIGURE 3:**
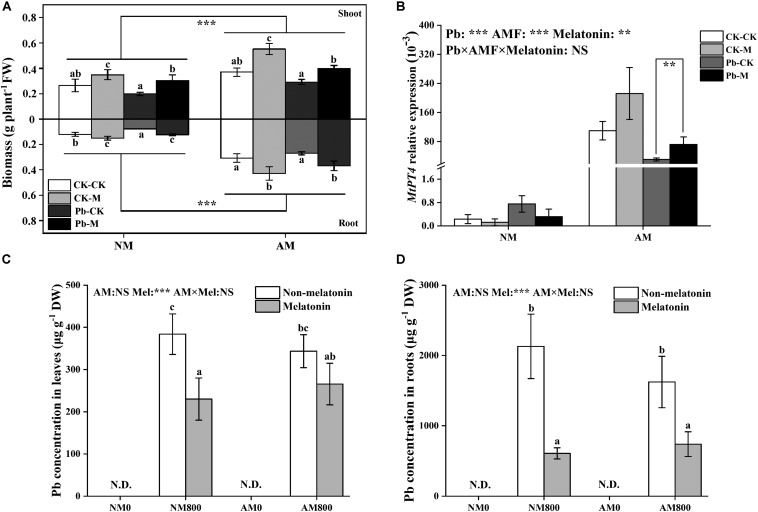
**(A)** The fresh weight both in shoots and roots of AM plants under Pb stress and melatonin application. The data are the means ± standard deviation (*n* = 4). Significant difference between NM plants and AM plants were labeled followed by the effect of two-way ANOVA. CK-CK = absence of both Pb stress and melatonin application; CK-M = melatonin application without Pb stress; Pb-CK = Pb stress without melatonin application; Pb-M = presence of both Pb stress and melatonin application. **(B)** The relative expression of *MtPT4* of AM roots under Pb stress and melatonin application. **(C,D)** The Pb content of AM plants under Pb stress and melatonin application. The data are the means ± standard deviation (*n* = 4). Different letters above the columns indicate significant difference among the means by Tukey HSD’s test (*P* < 0.05). **P < 0.01, ***P < 0.001; NS, not significant. N.D. = not detect. Mel = melatonin application. The abbreviation is consistent with the above.

Arbuscular mycorrhizal inoculation increased the shoot and root biomass of *M. truncatula* (Figure 3A) compared with the shoot and root biomass of NM plants with and without Pb stress. Melatonin application dramatically increased the shoot and root biomass. Pb stress significantly decreased the biomass of leaves and roots compared to unstressed plants.

The effect of AM colonization was positively enhanced by melatonin application with and without Pb stress (Table 1). AM plants to which melatonin was applied had the highest colonization of up to 92%. Pb stress decreased colonization with and without melatonin application.

**TABLE 1:** The AM colonization of *M. truncatula* under Pb stress and melatonin application.

Treatment	Colonization (%)
AM0	83.70 ± 2.87b
AM0M	91.66 ± 2.01c
AM800	74.33 ± 4.61a
AM800M	82.46 ± 3.87b
Significance of melatonin	**
Significance of Pb	***

The data are the means ± standard deviation (n = 4). The abbreviation is consistent with the above. **P < 0.01, ***P < 0.001.

### The Transcription of *MtPT4*

Arbuscular mycorrhizal inoculation largely increased (*P* < 0.05) the transcription of *MtPT4* with and without Pb stress. The relative expression of *MtPT4* in AM roots was decreased (*P* < 0.05) by Pb stress (Figure 3B). Melatonin application has a positive effect (*P* < 0.05) on *MtPT4* transcription under Pb stress.

### The Pb Concentration

Melatonin application decreased the root Pb concentration with and without AM inoculation (Figure 3D). With melatonin application, AM inoculation did not affect the Pb concentration in leaves and roots (Figures 3C,D). Without melatonin application, AM inoculation did not affect the root and leaf Pb concentration.

### Antioxidant Response

**FIGURE 4:**
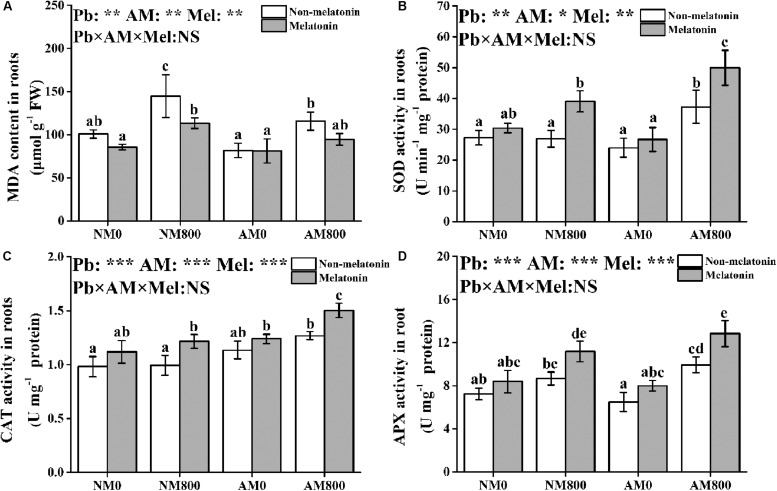
**(A–D)** The MDA contents, SOD activity, CAT activity, and APX activity of AM roots under Pb stress and melatonin application. The data are the means ± standard deviation (*n* = 4). Different letters above the columns indicate significant difference among the means of MDA content or antioxidant enzyme activity by Tukey HSD’s test (*P* < 0.05), *P < 0.05, **P < 0.01, ***P < 0.001; NS, not significant. The abbreviation is consistent with the above.

Pb stress significantly increased MDA content in roots compared to the roots of unstressed plants (Figure 4A). AM inoculation significantly decreased the MDA content in the roots of plants under Pb stress. Melatonin application decreased the MDA content in AM and NM plants under Pb stress but did not affect MDA content without Pb stress.

In roots, AM inoculation and melatonin application did not affect SOD activity in the absence of Pb stress (Figure 4B). AM inoculation and melatonin application increased the SOD, CAT, and APX activities in roots under Pb stress (Figures 4B–D). Pb stress increased the CAT activity in AM roots. AM roots under Pb stress to which melatonin was applied had the highest SOD, CAT, and APX activities among all treatments.

### Proline Content and Synthesis

**FIGURE 5:**
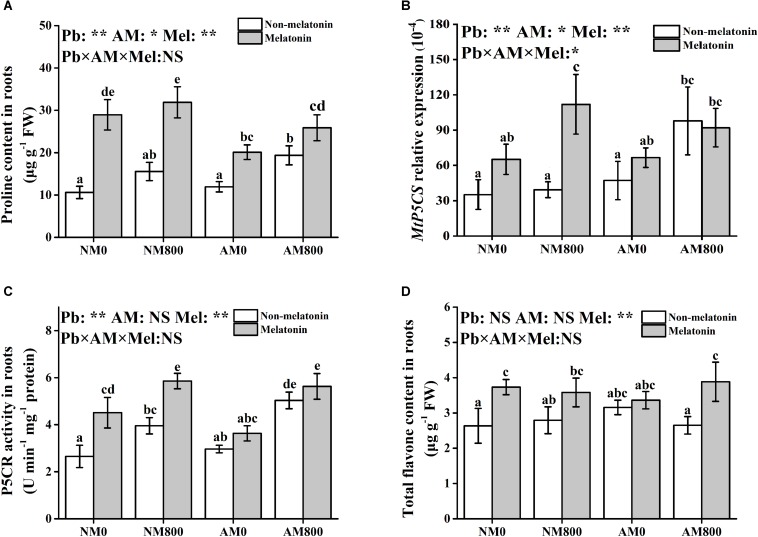
**(A–D)** The proline content, *MtP5CS* expression, P5CR activity, and total flavone content of AM roots under Pb stress and melatonin application. The data are the means ± standard deviation (*n* = 4). Different letters above the columns indicate significant difference among the means by Tukey HSD’s test (*P* < 0.05), *P < 0.05, **P < 0.01; NS, not significant. The abbreviation is consistent with the above.

In roots, melatonin application increased the proline content and P5CR activity compared to plants not treated with melatonin (Figures 5A,C). However, melatonin application did not affect the transcription of *MtP5CS* in AM roots (Figure 5B). Melatonin application upregulated the expression of *MtP5CS* in NM roots with and without Pb stress. Without melatonin application, AM inoculation increased the proline content and P5CR activity. However, with melatonin application, NM roots had a higher proline content than AM roots with and without Pb stress. Pb stress increased the root proline content and P5CR activity.

### Total Flavone Content

In NM plants, melatonin application increased the total flavone content with Pb stress (Figure 5D). In AM plants under Pb stress, melatonin application increased the total flavone content. Pb stress and AM inoculation did not affect the root flavone content.

## Discussion

### Arbuscular Mycorrhizal Inoculation Enhanced Melatonin Synthesis Under Pb Stress

Melatonin helps plants cope with oxidative damage through plant signal transduction and play the role of an endogenous antioxidant (Manchester et al., 2015; Zhan et al., 2019). In this study, Pb stress stimulated the accumulation of melatonin in roots, indicating that Pb-stressed plants may increase their antioxidant capacity by increasing melatonin accumulation. Several abiotic stresses, such as high temperature (Byeon and Back, 2014) and chemical stress (Arnao and HernándezRuiz, 2010), increased melatonin content in plants, which also indicated that melatonin accumulation is a positive response to abiotic stress. AM inoculation promoted melatonin synthesis under Pb stress. This result suggested that AM plants can become melatonin-rich under Pb stress to cope with Pb toxicity. Endophytic bacteria from grapevine roots could produce melatonin in response to abiotic stress (Jiao et al., 2016). Liu et al. (2016) observed melatonin accumulation in *Trichoderma asperellum* fungus under stress, which indicated that microorganisms could participate in melatonin accumulation under abiotic stress. Therefore, Melatonin produced by AM fungi may contribute to melatonin accumulation in *M. truncatula* roots under Pb stress. In plants, the melatonin accumulation is dependent on the efficiency of the synthesis of melatonin from serotonin (Kang et al., 2013; Byeon et al., 2014). In sunflower roots, salt stress increased the melatonin content by upregulating ASMT activity, revealing the positive effect of ASMT on melatonin synthesis (Mukherjee et al., 2014). Correlation analysis revealed a significant positive correlation between the relative expression of *MtASMT* and melatonin content. Kang et al. (2013) suggested that ASMT rather than SNAT is the rate-limiting enzyme in plant melatonin synthesis. Cd-stressed rice showed increased melatonin accumulation due to downregulated *SNAT* expression and upregulated *ASMT* expression (Byeon et al., 2015). Therefore, AM inoculation may induce melatonin synthesis in roots by stimulating the expression of *MtASMT*. AM plants are more suitable to survive from Pb stress than NM plants due to better melatonin regulation.

### Melatonin Application Improved AM Symbiosis by Decreasing Pb Accumulation

When host plants are exposed to Pb-contaminated environments, the high level of Pb inhibits protein activity, alters membrane oxidation, and disturbs mineral nutrient uptake (Nagajyoti et al., 2010). In this study, both AM inoculation and melatonin application increased *M. truncatula* growth under Pb stress, which suggested that both AM inoculation and melatonin can improve Pb stress tolerance. The adverse effects of Pb toxicity are usually caused by the excessive uptake of Pb in host plants. Once Pb has fixed to root surface, Pb could penetrate the root system passively and follows the water conduction system (Pourrut et al., 2013). In watermelon seedlings, melatonin treatment enhances tolerance to vanadium stress by decreasing the vanadium content and stimulating the antioxidant response (Nawaz et al., 2018). In addition, melatonin application reduced cadmium uptake and mitigated cadmium toxicity in tomato plants (Li et al., 2016). Gu et al. (2017) suggested that reduced cadmium uptake may be caused by melatonin-modulated heavy metal transporters. Melatonin inhibited Pb uptake under Pb stress, which may alleviate Pb toxicity. Taken together, these results suggest that melatonin application improves AM plant growth by the inhibition of Pb absorption.

MtPT4 is a low-affinity phosphate (Pi) transporter in *M. truncatula* located in arbuscule-colonized cells (Javot et al., 2007). *MtPT4* expression is induced by AM symbiosis, and *MtPT4* is usually used to characterize the relationship between AM fungi and host plant (Javot et al., 2007; Hu et al., 2017). In this study, Pb stress decreased AM colonization and inhibited *MtPT4* expression, which suggests Pb accumulation in roots has a negative effect on AM fungal inoculation. In *Lycium barbarum*, AM inoculation can maintain the ability of phosphate transporters to cope with drought stress (Hu et al., 2017). In alfalfa, AM plants upregulated the expression of *MsPT4* and *MsMT2* to decrease arsenic uptake and increase phosphorus uptake (Li et al., 2018). Melatonin-treated plants have lower Pb accumulation in root and lower Pb toxicity to the symbiosis structure. Therefore, melatonin-treated plants enhanced AM colonization by upregulating *MtPT4* expression and decreasing Pb accumulation. The synergistic effect of melatonin and AM symbiosis on plant growth may be because melatonin treatment resulted in a tighter AM symbiotic relationship.

### Melatonin Application Induced Antioxidant Response in Mycorrhizal Plants

The main reason for the negative effect of Pb stress on plant growth is the induction of oxidative injury by excessive Pb in plants. Pb stress affects respiration and blocks the leakage of electron transport chain (Shahid et al., 2012; Kohli et al., 2017), which induces the production of reactive oxygen species (ROS). The MDA content was measured to evaluate the levels of oxidative damage in plants (Fu and Huang, 2001). Both melatonin application and AM inoculation protected *M. truncatula* from oxidative injury by decreasing the MDA content. The O_2_^–^/H_2_O_2_ system, which converts ROS into non-toxic molecules, such as H_2_O, by enzymatic reactions, is one of the key elements in the response to oxidative injury. The SOD activity was increased by melatonin application and AM inoculation under Pb stress, which indicated that AM plants to which melatonin was applied have an increased ability to scavenge O_2_^–^ radicals (Baxter et al., 2013). Excessive H_2_O_2_ in the roots was detoxified by CAT or the AsA-GSH cycle. Melatonin application and AM treatment increased the CAT and APX activities under Pb stress, which indicated that melatonin application and AM inoculation are beneficial for H_2_O_2_ cleavage. Therefore, the synergistic effect of melatonin and AM symbiosis on Pb tolerance may be due to decreased oxidative injury and increased antioxidant enzyme activity.

Finally, several protective molecules, such as proline and flavonoids, participate in heavy metal detoxification (Chadzinikolau et al., 2017; Lima et al., 2019). Melatonin application promoted the accumulation of proline and flavonoids under Pb stress, which indicated that melatonin application enhances Pb detoxification in both AM and NM roots. In addition, melatonin application increased proline synthesis via increasing P5CR activity and the relative expression of *MtP5CS* (Stein et al., 2011). In *M. sativa*, melatonin application improved drought damage by increasing proline accumulation and synthesis pathway (Antoniou et al., 2017). Liang et al. (2018) suggested that melatonin application increases antioxidant capacity by increasing SOD, CAT, and POD activity and alleviates leaf senescence by promoting flavonoid biosynthesis. Melatonin application increased phenylalanine ammonia-lyase (PAL) activity to tolerant *Marssonina* apple blotch (Yin et al., 2013; Abdallah et al., 2016), suggesting the potential role of melatonin in flavonoid synthesis. Therefore, melatonin application increases antioxidant capacity and Pb detoxification by stimulating the antioxidant response and increasing the accumulation of protective molecules. However, AM inoculation and melatonin application did not have a synergistic effect on proline and flavonoid accumulations. This finding suggested that AM fungi and melatonin application use different methods to increase Pb tolerance. The synthesis of both flavonoids and proline is regulated by salicylic acid (Misra and Saxena, 2009; Abdallah et al., 2016). The opposite effects of melatonin application (Park et al., 2013) and AM inoculation (Medina et al., 2003) on salicylic acid regulation may account for this result. Certainly, further study should be conducted to determine whether salicylic acid metabolism leads to the interaction between AM symbiosis and melatonin under abiotic stress.

## Conclusion

To the best of our knowledge, this study is the first to analyze the effect of melatonin on heavy metal stress tolerance in AM plants, providing a new mechanism of Pb tolerance. AM inoculation may stimulate the accumulation of melatonin through the upregulation of *MtASMT* in roots. Melatonin application may inhibit Pb uptake to improve AM symbiosis under Pb stress. AM inoculation and melatonin application had a synergistic effect on host plant growth and Pb stress tolerance. This synergy may be due to improved AM symbiosis, alleviated the oxidative injury, and increased antioxidant enzyme activity. Overall, our results suggest that melatonin application could enhance mycorrhizal plant growth and Pb stress tolerance through stimulating antioxidant response and improving AM symbiosis. The combined use of AM inoculation and melatonin treatment is a potential way to help host plants cope with heavy metal toxicity.

## Data Availability Statement

All datasets generated for this study are included in the article/Supplementary Material.

## Author Contributions

XZ designed and conducted the study, harvested the samples, collected the data, and wrote the manuscript. HuZ conducted the study and collected the data. HaZ polished the manuscript. MT proposed the research and managed the funding.

## Conflict of Interest

The authors declare that the research was conducted in the absence of any commercial or financial relationships that could be construed as a potential conflict of interest.

## References

[B1] Abdallah S. B. Aung B. Amyot L. Lalin I. Lachâal M. Karray-Bouraoui N. (2016). Salt stress (NaCl) affects plant growth and branch pathways of carotenoid and flavonoid biosyntheses in *Solanum nigrum*. *Acta Physiol. Plant.* 38:72 10.1007/s11738-016-2096-8

[B2] Antoniou C. Chatzimichail G. Xenofontos R. Pavlou J. J. Panagiotou E. Christou A. (2017). Melatonin systemically ameliorates drought stress-induced damage in *Medicago sativa* plants by modulating nitro-oxidative homeostasis and proline metabolism. *J. Pineal Res.* 62:e12401. 10.1111/jpi.12401 28226194

[B3] Arnao M. B. HernándezRuiz J. (2010). Chemical stress by different agents affects the melatonin content of barley roots. *J. Pineal Res.* 46 295–299. 10.1111/j.1600-079X.2008.00660.x 19196434

[B4] Arnao M. B. Hernandez-Ruiz J. (2019). Melatonin: a new plant hormone and/or a plant master regulator? *Trends Plant Sci.* 24 38–48. 10.1016/j.tplants.2018.10.010 30446305

[B5] Aroca R. Ruiz-Lozano J. M. Zamarreño Á. M. Paz J. A. García-Mina J. M. Pozo M. J. (2013). Arbuscular mycorrhizal symbiosis influences strigolactone production under salinity and alleviates salt stress in lettuce plants. *J. Plant Physiol.* 70 47–55. 10.1016/j.jplph.2012.08.020 23102876

[B6] Back K. Tan D. X. Reiter R. J. (2016). Melatonin biosynthesis in plants: multiple pathways catalyze tryptophan to melatonin in the cytoplasm or chloroplasts. *J. Pineal Res.* 61 426–437. 10.1111/jpi.12364 27600803

[B7] Bates L. S. Waldren R. P. Teare I. D. (1973). Rapid determination of free proline for water-stress studies. *Plant Soil* 39 205–207. 10.1007/BF00018060 20688380

[B8] Baxter A. Mittler R. Suzuki N. (2013). ROS as key players in plant stress signaling. *J. Exp. Bot.* 65 1229–1240. 10.1093/jxb/ert375 24253197

[B9] Beyer W. F. Jr. Fridovich I. (1987). Assaying for superoxide dismutase activity: some large consequences of minor changes in conditions. *Anal. Biochem.* 161 559–566. 10.1016/0003-2697(87)90489-1 3034103

[B10] Burkhardt S. Tan D. X. Manchester L. C. Hardeland R. Reiter R. J. (2001). Detection and quantification of the antioxidant melatonin in Montmorency and Balaton tart cherries (*Prunus cerasus*). *J. Agric. Food Chem.* 49 4898–4902. 10.1021/jf010321 11600041

[B11] Byeon Y. Back K. (2014). Melatonin synthesis in rice seedlings in vivo is enhanced at high temperatures and under dark conditions due to increased serotonin N-acetyltransferase and N- acetylserotonin methyltransferase activities. *J. Pineal Res.* 56 189–195. 10.1111/jpi.12111 24313332

[B12] Byeon Y. Lee H. Y. Hwang O. J. Lee H. J. Lee K. Back K. (2015). Coordinated regulation of melatonin synthesis and degradation genes in rice leaves in response to cadmium treatment. *J. Pineal Res.* 58 470–478. 10.1111/jpi.12232 25783167

[B13] Byeon Y. Lee H. Y. Lee K. Back K. (2014). Caffeic acid O-methyltransferase is involved in the synthesis of melatonin by methylating N-acetylserotonin in *Arabidopsis*. *J. Pineal Res.* 57 219–227. 10.1111/jpi.12160 25039887

[B14] Chadzinikolau T. Kozłowska M. Mleczek M. (2017). Induction of phytochelatins and flavonoids in cadmium polluted *Berberis thunbergii*. *Dendrobiology* 77 139–146. 10.12657/denbio.077.011

[B15] Dubbels R. Reiter R. J. Klenke E. Goebel A. Schnakenberg E. Ehlers C. (1995). Melatonin in edible plants identified by radioimmunoassay and by high performance liquid chromatography-mass spectrometry. *J. Pineal Res.* 18 28–31. 10.1111/j.1600-079x.1995.tb00136.x 7776176

[B16] Fu J. Huang B. (2001). Involvement of antioxidants and lipid peroxidation in the adaptation of two cool-season grasses to localized drought stress. *Environ. Exp. Bot.* 45 105–114. 10.1016/s0098-8472(00)00084-8 11275219

[B17] Gu Q. Chen Z. Yu X. Cui W. Pan J. Zhao G. (2017). Melatonin confers plant tolerance against cadmium stress via the decrease of cadmium accumulation and reestablishment of microRNA-mediated redox homeostasis. *Plant Sci.* 261 28–37. 10.1016/j.plantsci.2017.05.001 28554691

[B18] Hu W. Zhang H. Zhang X. Chen H. Tang M. (2017). Characterization of six PHT1 members in *Lycium barbarum* and their response to arbuscular mycorrhiza and water stress. *Tree Physiol.* 37 351–366. 10.1093/treephys/tpw125 28062728

[B19] Isayenkov S. Fester T. Hause B. (2004). Rapid determination of fungal colonization and arbuscule formation in roots of *Medicago truncatula* using real-time (RT) PCR. *J. Plant Physiol.* 161 1379–1383. 10.1016/j.jplph.2004.04.012 15658808

[B20] Javot H. Penmetsa R. V. Terzaghi N. Cook D. R. Harrison M. J. (2007). A *Medicago truncatula* phosphate transporter indispensable for the arbuscular mycorrhizal symbiosis. *Proc. Natl. Acad. Sci. U.S.A.* 104 1720–1725. 10.1073/pnas.0608136104 17242358 PMC1785290

[B21] Jiang Y. Xie Q. Wang W. Yang J. Zhang X. Yu N. (2018). *Medicago* AP2-domain transcription factor *WRI5a* is a master regulator of lipid biosynthesis and transfer during mycorrhizal symbiosis. *Mol. Plant* 11 1344–1359. 10.1016/j.molp.2018.09.006 30292683

[B22] Jiao J. Ma Y. Chen S. Liu C. Song Y. Qin Y. (2016). Melatonin-producing endophytic bacteria from grapevine roots promote the abiotic stress-induced production of endogenous melatonin in their hosts. *Front. Plant Sci.* 7:1387. 10.3389/fpls.2016.01387 27708652 PMC5030213

[B23] Kang K. Lee K. Park S. Byeon Y. Back K. (2013). Molecular cloning of rice serotonin N- acetyltransferase, the penultimate gene in plant melatonin biosynthesis. *J. Pineal Res.* 55 7–13. 10.1111/jpi.12011 22998587

[B24] Kohli S. K. Handa N. Sharma A. Kumar V. Kaur P. Bhardwaj R. (2017). Synergistic effect of 24-epibrassinolide and salicylic acid on photosynthetic efficiency and gene expression in *Brassica juncea* L. under Pb stress. *Turk. J. Biol.* 41 943–953. 10.3906/biy-1707-15 30814859 PMC6353293

[B25] Kumar G. Knowles N. R. (1993). Changes in lipid peroxidation and lipolytic and free-radical scavenging enzyme activities during aging and sprouting of potato (*Solanum tuberosum*) seed-tubers. *Plant Physiol.* 102 115–124. 10.1104/pp.102.1.115 12231802 PMC158753

[B26] Lee K. Choi G. H. Back K. (2017). Cadmium-induced melatonin synthesis in rice requires light, hydrogen peroxide, and nitric oxide: key regulatory roles for tryptophan decarboxylase and caffeic acid O-methyltransferase. *J. Pineal Res.* 63:e12441. 10.1111/jpi.12441 28793366

[B27] Lerner A. B. Case J. D. Takahashi Y. (1960). Isolation of melatonin and 5-methoxyindole-3-acetic acid from bovine pineal glands. *J. Biol. Chem.* 235 1992–1997. 10.1002/jbmte.390020410 14415935

[B28] Li J. Sun Y. Jiang X. Chen B. Zhang X. (2018). Arbuscular mycorrhizal fungi alleviate arsenic toxicity to *Medicago sativa* by influencing arsenic speciation and partitioning. *Ecotoxicol. Environ. Saf.* 157 235–243. 10.1016/j.ecoenv.2018.03.073 29625397

[B29] Li M. Q. Hasan M. K. Li C. X. Ahammed G. J. Xia X. J. Shi K. (2016). Melatonin mediates selenium-induced tolerance to cadmium stress in tomato plants. *J. Pineal Res.* 61 291–302. 10.1111/jpi.12346 27264631

[B30] Li Z. Ma Z. Van der Kuijp T. J. Yuan Z. Huang L. (2014). A review of soil heavy metal pollution from mines in China: pollution and health risk assessment. *Sci. Total Environ.* 468–469 843–853. 10.1016/j.scitotenv.2013.08.090 24076505

[B31] Liang D. Shen Y. Ni Z. Wang Q. Lei Z. Xu N. (2018). Exogenous melatonin application delays senescence of kiwifruit leaves by regulating the antioxidant capacity and biosynthesis of flavonoids. *Front. Plant Sci.* 9:426. 10.3389/fpls.2018.00426 29675031 PMC5896581

[B32] Lima L. W. Checchio M. V. dos Reis A. R. de Cássia Alves R. Tezzoto T. Gratão P. L. (2019). Selenium restricts cadmium uptake and improve micronutrients and proline concentration in tomato fruits. *Biocatal. Agric. Biotechnol.* 18:101057 10.1016/j.bcab.2019.101057

[B33] Liu T. Zhao F. Liu Z. Zuo Y. Hou J. Wang Y. (2016). Identification of melatonin in *Trichoderma* spp. and detection of melatonin content under controlled-stress growth conditions from *T. asperellum*. *J. Basic Microbiol.* 56 838–843. 10.1002/jobm.201500223 26367376

[B34] Madan S. Nainawatee H. S. Jain R. K. Chowdhury J. B. (1995). Proline and proline metabolising enzymes in in-vitro selected nacl-tolerant *Brassica juncea* L. under salt stress. *Ann. Bot.* 76 51–57. 10.1006/anbo.1995.1077

[B35] Manchester L. C. Coto-Montes A. Boga J. A. Andersen L. P. H. Zhou Z. Galano A. (2015). Melatonin: an ancient molecule that makes oxygen metabolically tolerable. *J. Pineal Res.* 59 403–419. 10.1111/jpi.12267 26272235

[B36] Medina M. J. H. Gagnon H. Piché Y. Ocampo J. A. Garrido J. M. G. Vierheilig H. (2003). Root colonization by arbuscular mycorrhizal fungi is affected by the salicylic acid content of the plant. *Plant Sci.* 164 993–998. 10.1016/s0168-9452(03)00083-9 31700111

[B37] Misra N. Saxena P. (2009). Effect of salicylic acid on proline metabolism in lentil grown under salinity stress. *Plant Sci.* 177 181–189. 10.1016/j.plantsci.2009.05.007

[B38] Mukherjee S. David A. Yadav S. Baluška F. Bhatla S. C. (2014). Salt stress-induced seedling growth inhibition coincides with differential distribution of serotonin and melatonin in sunflower seedling roots and cotyledons. *Physiol. Plant.* 152 714–728. 10.1111/ppl.12218 24799301

[B39] Nagajyoti P. C. Lee K. D. Sreekanth T. V. M. (2010). Heavy metals, occurrence and toxicity for plants: a review. *Environ. Chem. Lett.* 8 199–216. 10.1007/s10311-010-0297-8

[B40] Nakano Y. Asada K. (1981). Hydrogen peroxide is scavenged by ascorbate-specific peroxidase in spinach chloroplasts. *Plant Cell Physiol.* 22 867–880. 10.1093/oxfordjournals.pcp.a076232

[B41] Nawaz M. A. Jiao Y. Chen C. Shireen F. Zheng Z. Imtiaz M. (2018). Melatonin pretreatment improves vanadium stress tolerance of watermelon seedlings by reducing vanadium concentration in the leaves and regulating melatonin biosynthesis and antioxidant-related gene expression. *J. Plant Physiol.* 220 115–127. 10.1016/j.jplph.2017.11.003 29172132

[B42] Pape C. Lüning K. (2006). Quantification of melatonin in phototrophic organisms. *J. Pineal Res.* 41 157–165. 10.1111/j.1600-079X.2006.00348.x 16879322

[B43] Park S. Byeon Y. Back K. (2013). Transcriptional suppression of tryptamine 5-hydroxylase, a terminal serotonin biosynthetic gene, induces melatonin biosynthesis in rice (*Oryza sativa* L.). *J. Pineal Res.* 55 131–137. 10.1111/jpi.12053 23521226

[B44] Pourrut B. Shahid M. Douay F. Dumat C. Pinelli E. (2013). “Molecular mechanisms involved in lead uptake, toxicity and detoxification in higher plants,” in *Heavy Metal Stress in Plants*, eds Gupta D. Corpas F. Palma J. (Berlin: Springer), 121–147. 10.1007/978-3-642-38469-1_7

[B45] Sánchez-Romera B. Ruiz-Lozano J. M. Zamarreño Á. M. García-Mina J. M. Aroca R. (2016). Arbuscular mycorrhizal symbiosis and methyl jasmonate avoid the inhibition of root hydraulic conductivity caused by drought. *Mycorrhiza* 26 111–122. 10.1007/s00572-015-0650-7 26070449

[B46] Shahid M. Dumat C. Silvestre J. Pinelli E. (2012). Effect of fulvic acids on lead-induced oxidative stress to metal sensitive *Vicia faba* L. plant. *Biol. Fert. Soils* 48 689–697. 10.1007/s00374-012-0662-9

[B47] Stein H. Honig A. Miller G. Erster O. Eilenberg H. Csonka L. N. (2011). Elevation of free proline and proline-rich protein levels by simultaneous manipulations of proline biosynthesis and degradation in plants. *Plant Sci.* 181 140–150. 10.1016/j.plantsci.2011.04.013 21683879

[B48] Tan D. X. Reiter R. J. (2015). Melatonin: an ancient molecule that makes oxygen metabolically tolerable. *J. Pineal Res.* 59 403–419. 26272235 10.1111/jpi.12267

[B49] Tang Y. Lin L. Xie Y. Liu J. Sun G. Li H. (2018). Melatonin affects the growth and cadmium accumulation of *Malachium aquaticum* and *Galinsoga parviflora*. *Int. J. Phytoremediation* 20 295–300. 10.1080/15226514.2017.1374341 29053350

[B50] Tüzen M. (2003). Determination of heavy metals in soil, mushroom and plant samples by atomic absorption spectrometry. *Microchem. J.* 74 289–297. 10.1016/S0026-265X(03)00035-3

[B51] Wang P. Zhang S. Wang C. Lu J. (2012). Effects of Pb on the oxidative stress and antioxidant response in a Pb bioaccumulator plant *Vallisneria natans*. *Ecotoxiol. Environ. Saf.* 78 28–34. 10.1016/j.ecoenv.2011.11.008 22138147

[B52] Wei J. Li D. X. Zhang J. R. Shan C. Rengel Z. Song Z. B. (2018). Phytomelatonin receptor PMTR 1-mediated signaling regulates stomatal closure in *Arabidopsis thaliana*. *J. Pineal Res.* 65:e12500. 10.1111/jpi.12500 29702752

[B53] Yabe J. Nakayama S. M. Ikenaka Y. Yohannes Y. B. Bortey-Sam N. Kabalo A. N. (2018). Lead and cadmium excretion in feces and urine of children from polluted townships near a lead-zinc mine in Kabwe, Zambia. *Chemosphere* 202 48–55. 10.1016/j.chemosphere.2018.03.079 29554507

[B54] Yang Q. Li Z. Lu X. Duan Q. Huang L. Bi J. (2018). A review of soil heavy metal pollution from industrial and agricultural regions in China: pollution and risk assessment. *Sci. Total Environ.* 642 690–700. 10.1016/j.scitotenv.2018.06.068 29909337

[B55] Yang Y. Song Y. Scheller H. V. Ghosh A. Ban Y. Chen H. (2015). Community structure of arbuscular mycorrhizal fungi associated with *Robinia pseudoacacia* in uncontaminated and heavy metal contaminated soils. *Soil Biol. Biochem.* 86 146–158. 10.1016/j.soilbio.2015.03.018

[B56] Yin L. Wang P. Li M. Ke X. Li C. Liang D. (2013). Exogenous melatonin improves Malus resistance to *Marssonina* apple blotch. *J. Pineal Res.* 54 426–434. 10.1111/jpi.12038 23356947

[B57] Zeng T. Rodriguez-Moreno L. Mansurkhodzaev A. Wang P. van den Berg W. Gasciolli V. (2020). A lysin motif effector subverts chitin-triggered immunity to facilitate arbuscular mycorrhizal symbiosis. *New Phytol.* 225 448–460. 10.1111/nph.16245 31596956 PMC6916333

[B58] Zhan H. Nie X. Zhang T. Li S. Wang X. Du X. (2019). Melatonin: a small molecule but important for salt stress tolerance in plants. *Int. J. Mol. Sci.* 20:709. 10.3390/ijms20030709 30736409 PMC6387279

[B59] Zhishen J. Mengcheng T. Jianming W. (1999). The determination of flavonoid contents in mulberry and their scavenging effects on superoxide radicals. *Food Chem.* 64 555–559. 10.1016/S0308-8146(98)00102-2

