# Supplementary material for: Exogenous Melatonin Application Enhances Rhizophagus irregularis Symbiosis and Induces the Antioxidant Response of Medicago truncatula Under Lead Stress
Source: Front Microbiol. 2020 Apr 15;11:516. doi: 10.3389/fmicb.2020.00516 (PMC7174712; doi:10.3389/fmicb.2020.00516)
Supplement: Supplementary file 1 [file Image_1.pdf]

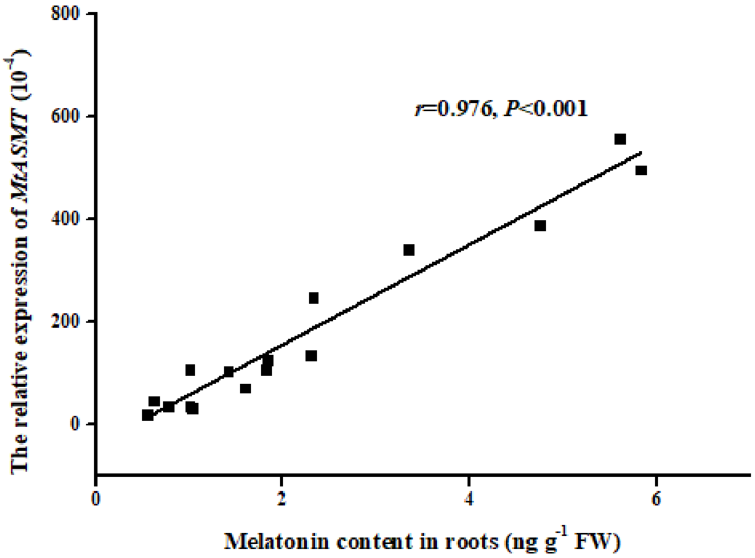

Supplementary Figure S1 Correlation analysis between the *MtASMT* expression and melatonin contents in roots. n=16
